# Higher Dietary Magnesium Intake and Higher Magnesium Status Are Associated with Lower Prevalence of Coronary Heart Disease in Patients with Type 2 Diabetes

**DOI:** 10.3390/nu10030307

**Published:** 2018-03-05

**Authors:** Christina M. Gant, Sabita S. Soedamah-Muthu, S. Heleen Binnenmars, Stephan J. L. Bakker, Gerjan Navis, Gozewijn D. Laverman

**Affiliations:** 1 Department of Internal Medicine/Nephrology, ZGT Hospital, 7609 PP Almelo, The Netherlands; c.gant@zgt.nl; 2 Department of Internal Medicine, Division of Nephrology, University of Groningen, University Medical Centre Groningen, 9713EZ Groningen, The Netherlands; s.h.binnenmars@umcg.nl (S.H.B.); s.j.l.bakker@umcg.nl (S.J.L.B.); g.j.navis@umcg.nl (G.N.); 3 Centre of Research on Psychology in Somatic Diseases (CORPS), Department of Medical and Clinical Psychology, Tilburg University, 5037 AB Tilburg, The Netherlands; S.S.Soedamah@uvt.nl; 4 Institute for Food, Nutrition and Health, University of Reading, Reading RG1 5EX, UK

**Keywords:** coronary heart disease, diabetes mellitus type 2, dietary magnesium intake, urinary magnesium excretion, plasma magnesium concentration

## Abstract

In type 2 diabetes mellitus (T2D), the handling of magnesium is disturbed. Magnesium deficiency may be associated with a higher risk of coronary heart disease (CHD). We investigated the associations between (1) dietary magnesium intake; (2) 24 h urinary magnesium excretion; and (3) plasma magnesium concentration with prevalent CHD in T2D patients. This cross-sectional analysis was performed on baseline data from the DIAbetes and LifEstyle Cohort Twente-1 (DIALECT-1, *n* = 450, age 63 ± 9 years, 57% men, and diabetes duration of 11 (7–18) years). Prevalence ratios (95% CI) of CHD by sex-specific quartiles of magnesium indicators, as well as by magnesium intake per dietary source, were determined using multivariable Cox proportional hazard models. CHD was present in 100 (22%) subjects. Adjusted CHD prevalence ratios for the highest compared to the lowest quartiles were 0.40 (0.20, 0.79) for magnesium intake, 0.63 (0.32, 1.26) for 24 h urinary magnesium excretion, and 0.62 (0.32, 1.20) for plasma magnesium concentration. For every 10 mg increase of magnesium intake from vegetables, the prevalence of CHD was, statistically non-significantly, lower (0.75 (0.52, 1.08)). In this T2D cohort, higher magnesium intake, higher 24 h urinary magnesium excretion, and higher plasma magnesium concentration are associated with a lower prevalence of CHD.

## 1. Introduction

Coronary heart disease (CHD) is one of the most prevalent and high-impact complications related to type 2 diabetes mellitus (T2D) [1,2]. In the general population, magnesium (Mg) deficiency might be associated with a greater risk of CHD; however, data on the inverse associations between Mg status and intake and CHD are inconsistent [3,4,5,6,7,8,9]. In T2D, the prevalence of hypomagnesemia is increased 14–48%, compared with 3–15% in those without T2D [10,11]. This could partly be due to increased urinary Mg excretion caused by insulin resistance, and partly due to poor dietary Mg intake [10,11,12,13]. However, surprisingly few studies report on the association between Mg and CHD in patients with established T2D [14,15].

In the DIAbetes and LifEstyle Cohort Twente (DIALECT), we collected extensive data on dietary Mg intake, 24 h urinary Mg excretion, and plasma Mg concentration. We aimed to study the association between the parameters of Mg (i.e., dietary Mg intake, 24 h urinary Mg excretion, and serum Mg concentration) and CHD. When we found that dietary Mg intake was inversely associated with CHD risk, we also explored whether there was an association between Mg intake from specific dietary sources (i.e., cereals, potatoes, etc.) and CHD risk.

## 2. Materials and Methods 

### 2.1. Study Design

This was a cross-sectional analysis performed in the DIAbetes and LifEstyle Cohort Twente-1 (DIALECT-1). The study population and study procedures have been described previously [16]. The study has been approved by the relevant institutional review boards (METC-Twente, NL57219.044.16; METC-Groningen, 1009.68020), is registered in the Netherlands Trial Register (NTR trial code 5855), and is performed according to the guidelines of good clinical practice and the declaration of Helsinki. 

### 2.2. Participants

All patients with T2D treated in the outpatient clinic of our hospital, aged 18+ years, were eligible for the study. Exclusion criteria were inability to understand the informed consent procedure, insufficient command of the Dutch language, or dialysis dependency. The inclusion flowchart has been described previously [16]. In total, 1082 patients were eligible for the study, of which 470 agreed to participate. The most important reasons for non-participation were no interest in the trial (*n* = 123), inability due to co-morbidity (*n* = 62), and no transport options (*n* = 58). After the baseline visit, 20 patients were excluded due to the fact that their diabetes diagnosis changed from type 2 diabetes to type 1 diabetes. Therefore, a total of 450 patients with type 2 diabetes were included in DIALECT-1. Missing values for specific variables are listed in Table 1.

### 2.3. Study Procedures

Eligible patients with type 2 diabetes were selected from the electronic patient file. At the clinic, sociodemographic characteristics, medical history, lifestyle behaviors, and current medications were recorded and anthropometric dimension were measured. Blood pressure was measured in a supine position by an automated device (Dinamap^®^; GE Medical systems, Milwaukee, WI, USA) for 15 min with a one-minute interval. The mean systolic and diastolic pressure of the final three measurements was used for further analysis. Physical activity was assessed using the Short QUestionnaire to ASses Health enhancing physical activity (SQUASH) questionnaire, which was previously validated and is commonly used in the Netherlands for population research [17]. 

Blood was drawn from venipuncture, for measurement of Mg and other variables relevant for diabetes. 24-h urine collections were performed as prescribed previously [16]. Samples of blood and urine were stored at −80 °C for later analysis. 

### 2.4. Magnesium Measurements

We calculated dietary Mg intake using a semi-quantitative food frequency questionnaire (FFQ), inquiring about the intake of 177 items during the last month, taking seasonal variations into account [18]. The FFQ was developed and validated at the Wageningen University, and has been updated several times. For each food item, the frequency was recorded in times per day, week, or month. The number of servings was expressed in natural units (e.g., a slice of bread or a whole apple) or household measures (e.g., cup or spoon). Both questionnaires were self-administered and filled in at home. The filled-in questionnaires were checked for completeness by a trained researcher, and inconsistent answers were verified with the patients. If the patient could not remember an exact number, the trained researcher approximated the intake as closely as possible during the interview. Dietary data was converted into daily nutrient intake using the Dutch Food Composition Table of 2013 [19]. We calculated the average intake of Mg by multiplying the frequency of consumption of each food item by its Mg content in the Dutch Food Composition Table of 2013 [19], and summing the amount of Mg across all food items. We calculated Mg intake from different food categories by multiplying the frequency of consumption of each food item in that specific category by its Mg content, and summing across all food items in that category. Food items of the FFQ included in each category are listed in supplementary Table S1.

Plasma and urinary Mg were measured in stored plasma samples in routine laboratory measurements using the xylidyl blue method. Buffer/Ethylenediaminetetraacetic acid was added to mask calcium. After incubation, xylidyl blue was added to form a purple complex with Mg. Mg concentration was determined by the photometric measurement of xylidyl blue extinction. The detection range for plasma Mg was 0.1–5 mmol/L, and for 24 h urinary Mg the range was 0.5–25 mmol/L. There were no patients with values outside of the detection ranges. Hypomagnesemia was defined as serum Mg concentration <0.70 mmol/L.

### 2.5. Main Study Outcome

Coronary heart disease (CHD) is defined as physician-diagnosed unstable angina pectoris or myocardial infarction, percutaneous coronary intervention, or a coronary artery bypass graft in the medical history. Medical history was checked for CHD during the interview at the baseline visit, and was later reviewed in the hospital electronic patient files on three different occasions, by three different physician researchers, who were unware of the magnesium data.

### 2.6. Statistics

All statistical analyses were performed using the Statistical Package for the Social Sciences (SPSS), version 23.0. Normally-distributed data are presented as mean ± standard deviation. Skewed variables are expressed as median (interquartile range). Dichotomous variables are presented in number and percentage. Dietary intake of Mg was adjusted for energy intake by the residual method [20].

Differences between T2D patients with and without CHD were determined using the Student *t* (normal distribution), Mann–Whitney U (skewed distribution), or Chi-Square (categorical variables) test. In order to examine parameters associated with magnesium intake, we divided the population into sex-specific quartiles of adjusted magnesium intake. Differences between the quartiles were assessed using one-way ANOVA (normal distribution), Kruskal–Wallis (skewed distribution), or Chi-Square (categorical variables) analysis.

Correlations between Mg parameters were assessed using Pearson’s correlation coefficient.

We calculated the prevalence ratio (95% CI) of CHD by sex-specific quartiles of (1) dietary Mg intake; (2) 24 h urinary Mg excretion; and (3) plasma Mg concentration, using multivariable Cox proportional hazard models, with the time to event set at 1 for all patients. The models were adjusted for the potential confounding of lifestyle parameters (BMI, smoking, alcohol, and physical activity) and nutritional intake (24 h urinary sodium excretion and 24 h urinary potassium excretion) [3,21]. There was a strong correlation between dietary calcium and Mg intake (R = 0.70), therefore we did not adjust for calcium intake in the final model. Effect modification was checked for gender, BMI, smoking, and alcohol, and no significant effect modification was found (*p* > 0.20 for all interaction terms). Sensitivity analyses were performed by excluding patients with diabetic kidney disease, and prevalence ratios were similar as in the primary analyses.

Additionally, we performed multivariable Cox proportional hazard models, and calculated the prevalence ratios of CHD for each 10 mg increment of dietary Mg intake from different sources (cereals, dairy, coffee, potatoes, meat, legumes and nuts, fruit, vegetables, and other). The models were adjusted for potential confounding of lifestyle parameters (age, BMI, smoking, alcohol, and physical activity) and Mg intake from the miscellaneous sources.

## 3. Results

In total, 450 patients with T2D were included in DIALECT-1. Baseline characteristics are shown in Table 1. In short, patients were 63 ± 9 years old, and the majority of the population was male (57%). The population represents T2D in secondary health care, with a median diabetes duration of 11 (7–18) years, and a high prevalence of diabetic nephropathy (42%). 

There were 100 (22%) CHD cases diagnosed in our population (Table 1). T2D patients with CHD were older (66 ± 7 vs. 62 ± 9 years, *p* < 0.001), were more often men (71% vs. 54%, *p* = 0.003), and more often had peripheral artery disease (44% vs. 20%, *p* < 0.001), and nephropathy (58% vs. 38%, *p* < 0.001) than patients without CHD. There were no differences in lifestyle parameters between those with and without CHD. Regarding pharmacological treatment, those with CHD more often used beta-blockers (77% vs. 37%, *p* < 0.001), and loop diuretics (33% vs. 14%, *p* < 0.001) than those without CHD. This was paralleled by a lower diastolic blood pressure (72 ± 10 mmHg vs. 75 ± 9 mmHg, *p* = 0.01) and heart rate (69 ± 11 beats/min vs. 75 ± 13 beats/min, *p* < 0.001) in patients with CHD. Systolic blood pressure was 136 ± 16 mmHg, and did not differ between the groups. Although patients with CHD more often used statins (86% vs. 73%, *p* = 0.006), serum LDL was similar in the groups (2.0 ± 0.7 mmol/L), and serum HDL cholesterol was lower in those with CHD (1.0 ± 0.3 mmol/L vs. 1.2 ± 0.4 mmol/L, *p* < 0.001) compared to those without CHD. 

Mean energy-adjusted dietary Mg intake was 305 ± 46 mg/day, and was lower in those with CHD (adjusted standardized beta = −0.14, *p* = 0.003). Mean 24 h urinary Mg excretion was 3.94 ± 2.05 mmol/24 h, and mean plasma Mg concentration was 0.77 ± 0.09 mmol/L; neither differed statistically significantly between those with and without CHD (Table 1). Hypomagnesemia (plasma Mg < 0.7 mmol/L) was present in 73 patients (17%), of which 11 patients (3%) had a plasma Mg of <0.6 mmol/L. 

Dietary Mg intake was significantly correlated with 24 h urinary Mg excretion (Pearson R = 0.24, *p* < 0.001), but not with plasma Mg (R = 0.02, *p* = 0.64). 24 h urinary Mg excretion was significantly correlated with plasma Mg (R = 0.13, *p* < 0.008).

Systolic blood pressure was lowest in the highest gender-specific quartile of energy-adjusted magnesium intake (4th quartile 133 ± 13 vs. 1st quartile 137 ± 17 mmHg; Supplementary Table S2), and the number of antihypertensive drugs used was lowest in this quartile as well (4th quartile 2 (0–3) vs. 2 (1–3) in other quartiles, *p* = 0.008). Serum HbA1c and cholesterol levels were similar in all Mg intake quartiles. There was a trend towards higher urinary potassium excretion, dietary calcium, fiber, protein, and carbohydrate intake, as well as a lower dietary intake of fat in each of the higher quartiles of magnesium intake.

### 3.1. Association between Dietary Magnesium Intake, 24 h Urinary Magnesium Excretion, Plasma Magnesium Concentration, and the Prevalence of Coronary Heart Disease

The highest quartile of Mg intake was significantly associated with a lower prevalence ratio (PR) of CHD than the lowest quartile of Mg intake (0.40 (0.20, 0.77); Table 2). When adjusting for age and lifestyle parameters (BMI, smoking, alcohol, and physical activity), the PR remained largely unchanged (0.42 (0.22, 0.82)). After adjustment for dietary intake of other micronutrients (total energy intake, sodium, and potassium), the PR became (0.40 (0.20, 0.79)), and the *p*-trend was 0.01. 

There was a similar trend towards a lower prevalence of CHD in the highest quartile of 24 h urinary Mg excretion, which was not statistically significant (PR 0.63 (0.33, 1.19)). After adjustment for lifestyle and nutritional factors, the PR remained similar (0.63 (0.32, 1.26)). Also, the highest quartile of plasma Mg concentration had a non-significant trend towards a lower prevalence of CHD (unadjusted PR 0.60 (0.31, 1.14), adjusted PR 0.62 (0.32, 1.20)). The PR ratios for dietary Mg intake, urinary Mg excretion and plasma Mg concentration did not change after further adjustment for other classic CHD risk factors, namely systolic blood pressure and LDL cholesterol (data not shown).

### 3.2. Analysis on Source of Magnesium Intake and Prevalence of CHD

**Figure 1:**
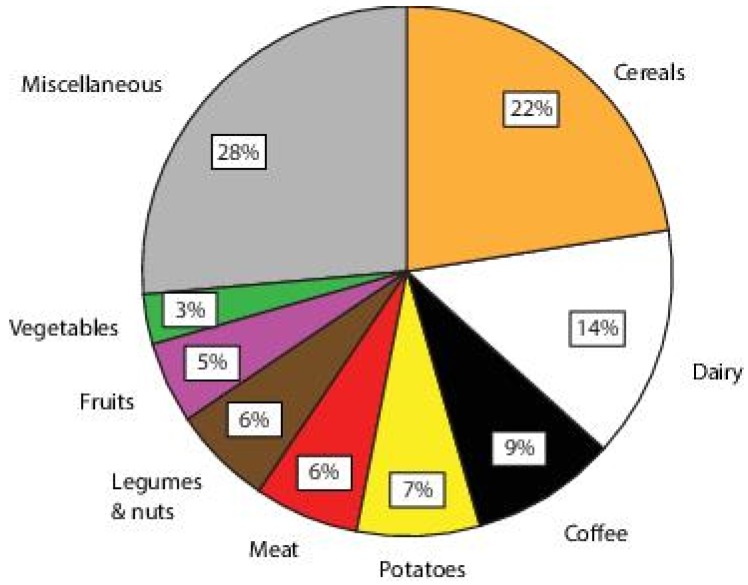
Sources of magnesium intake from different food product categories in patients with T2D.

We performed an explorative analysis whether there was an association between Mg intake from specific dietary sources and CHD. The largest dietary contributors to total dietary Mg intake for patients with T2D (Figure 1) were cereals at 22% (16–26%), dairy at 14% (10–20%), coffee at 9% (6–13%), potatoes at 7% (4–10%), meat at 6% (5–8%), legumes and nuts at 6% (4–11%), fruit at 5% (3–8%), and vegetables with 3% (2–5%). We found no statistically significant association between Mg intake from specific food groups and CHD (Table 3). However, there was a non-significant trend towards a lower prevalence of CHD for every 10 mg increase of dietary Mg intake derived from vegetables (PR 0.75 (0.52, 1.08)).

## 4. Discussion

We found inverse associations for dietary Mg intake, 24 h urinary Mg excretion, and plasma Mg concentration with the prevalence of CHD in patients with T2D. As far as we know, this is the first study in T2D patients which simultaneously reports on these three Mg parameters in relation to CHD. The inverse association between dietary Mg intake and the prevalence of CHD we found was strongest for Mg intake derived from vegetables, albeit not statistically significant. 

The mean dietary Mg intake we report (305 ± 46 mg/day) was somewhat lower than the median Mg intake in the general Dutch population, which is around 350 mg/day [22], but was comparable to median Mg intake of population studies in the U.S. (308 mg/day) [4]. We found that in the Dutch population, cereals, dairy, and coffee intake were the largest contributors to total Mg intake, at 22%, 14%, and 9% respectively. This was somewhat different from the U.K. population, where cereals (34%), meat (19%), and dairy (18%) intake were the most important contributors [23]. In contrast, in the U.S. population the most important food groups were vegetables (13%), milk (8%), and meat (7%) [24]. It should be noted that different groupings of food products renders a head-to-head comparison between these percentages difficult. The 24 h urinary Mg excretion we report (4.0 ± 2.1 mmol/24 h) was in line with the general population the Netherlands (4.2 ± 1.7 mmol/24 h for men and 3.5 ± 1.4 mmol/24 h for women) [5]. Plasma Mg concentration (0.77 ± 0.09 mmol/L) was similar to an earlier report about Dutch diabetes patients (0.74 ± 0.10 mmol/L) [25]. The prevalence of hypomagnesemia we found (17%) was in the range of the reported prevalence of hypomagnesemia in patients with T2D, between 14% and 48% [10,11], and emphasizes that clinical vigilance for hypomagnesemia is warranted in T2D, because it is associated with increased insulin resistance and faster renal function decline [26].

We are the first to report an inverse association between dietary Mg intake and the prevalence of CHD in T2D. In contrast, a large meta-analysis in non-T2D patients demonstrated no association between dietary Mg intake and incident CHD [9]. However, low dietary Mg intake was associated with a higher risk of stroke, heart failure, new-onset diabetes, and all-cause mortality [9]. It is known that in T2D renal wasting of Mg occurs [27]. Additionally, Mg supplementation in T2D can improve insulin sensitivity and metabolic control [28]. Possibly, an adequate Mg intake in patients with T2D is even more important than in those without T2D, in order to maintain an adequate Mg status and prevent diabetes-related complications. These data fuel the hypothesis that magnesium intake is beneficial in T2D patients.

In addition, when investigating Mg intake from specific food sources, we found the strongest inverse association between Mg derived from vegetables and CHD, albeit not quite reaching statistical significance. To our knowledge, the association with vegetable-derived Mg intake and CHD has not been described before. When studying Mg intake and Mg status, it is important to consider bioavailability of ingested Mg for intestinal uptake, as this might vary considerably depending on the source of Mg intake [29]. Possibly, bioavailability from Mg in vegetables is greater than from other food sources; however, this issue would have to be addressed an in-depth mechanistic study. Nevertheless, our data illustrate that when studying the association between micronutrients and outcomes, intake of different food groups is also important. As vegetable intake in our population was low [30], and vegetable intake only accounted for 3% of total Mg intake in this population, increasing vegetable intake is a good opportunity to not only increase Mg intake, but also to improve overall diet quality. It should be noted that in our study, it is difficult to distinguish between the protective effects of overall vegetable intake and those from vegetable-derived Mg intake. Other vegetable-derived components like antioxidants, but also potassium and vitamin K, might contribute to or interact with Mg in the eventual association with CHD [31,32,33,34]. Maybe the possible cohort effect from these micronutrients and Mg could amplify such protection. Since such an analysis is beyond the scope and available data of the current study, future studies are necessary to further investigate mechanisms behind vegetable intake and risk of coronary heart disease. 

Additionally, we found that lower 24 h urinary Mg excretion was associated with more prevalent CHD. In line with this finding, in the general population an inverse association between Mg excretion and CHD was reported [5]. Potential renal Mg wasting in T2D renders the interpretation of urinary Mg excretion difficult. High urinary Mg excretion could, on the one hand, reflect a high dietary Mg intake; on the other hand, though, it could reflect the hypermagnesuria found in T2D [10,11]. This could explain why, in our cohort, dietary Mg intake is more strongly associated with CHD than 24 h urinary excretion.

In parallel, lower plasma Mg concentration was also associated with prevalent CHD. In T2D, the association between plasma Mg concentration and CHD was investigated previously, and conflicting results were reported [14,15]. In non-T2D subjects, conflicting results on the association between plasma Mg have been reported as well; however, a meta-analysis demonstrated an inverse association between plasma Mg and incident CHD [8]. As Mg is mainly an intracellular cation, and therefore plasma Mg only reflects 1% of bodily Mg stores, the validity of using plasma Mg as a marker for Mg status has been questioned; Mg deficiency has been reported in patients without overt hypomagnesemia [11,35]. 

Our paper was not designed to unravel mechanisms behind the inverse associations between Mg intake, Mg status, and CHD. However, several mechanisms have been proposed that could underlie this association. First, animal studies have consistently shown that higher Mg status inhibits vascular calcification [36,37]. In human subjects, serum Mg and dietary Mg intake were inversely associated with the degree of coronary calcification [4,6]. Second, low Mg status might be associated with cardiac arrhythmias [38]. Lastly, increased CHD risk might be mediated through the association between low Mg status or intake and increased traditional CHD risk factors, such as blood pressure [39] and insulin resistance [12].

Our paper is the first to simultaneously report the association between dietary Mg intake, factors of Mg status (24 h urinary Mg excretion and plasma Mg), and CHD in patients with established T2D. The robustness of our findings is established through the fact that all three Mg parameters were inversely associated with CHD. The main limitation of our paper is the cross-sectional design, which only allowed us to study associations and not causality, and therefore there is a risk of reverse causality bias. Another limitation is that the FFQ we used in our study was not validated to estimate magnesium intake. However, because there was a moderate correlation between dietary Mg intake and urinary Mg excretion, we deemed the results sufficiently valid. 

Our study has several clinical implications. First, we show that Mg intake is of the utmost importance with relation to T2D. Patients with T2D are at risk of developing hypomagnesemia, as Mg intake in our population was somewhat lower in comparison to the general population. Additionally, patients with T2D have increased renal Mg excretion [27]. We show that Mg intake and Mg status is reduced in those with CHD, possibly indicating that higher Mg intake is associated with a lower risk of CHD. The best opportunity to increase Mg intake is to increase intake of Mg-rich vegetables. As Mg intake in the highest quartile is approximately 100 mg/day higher than in the lowest quartile, in clinical practice this could correspond with increasing vegetable intake by, for example, 200 g spinach, or 100 g rucola lettuce and two avocados per day. Alternatively, previous research has shown that several dietary patterns might reduce CHD risk or improve cardiac function, such as the Mediterranean diet; the Dietary Approaches to Stop Hypertension diet; or a high-protein, intermittent fasting, low-calorie diet [40,41,42]. We add to these findings by illustrating that Mg is an important component in such diets. For future studies, it would be of interest to investigate how Mg and other beneficial nutritional approaches could reinforce each other in the pursuit of the reduction of CHD in diabetes patients. Additionally, future mechanistic studies should be done to investigate how vegetable-derived nutrients, particularly Mg, might reduce CHD risk.

## 5. Conclusions

In a cohort of patients with established T2D, dietary magnesium intake, 24 h urinary magnesium excretion, and plasma magnesium concentration were inversely associated with the prevalence of coronary heart disease. Increasing dietary magnesium intake, especially through increasing vegetable intake, may reduce the risk of CHD in patients with established T2D.

## Figures and Tables

**Table 1:** Baseline characteristics of patients with type 2 diabetes mellitus (T2D) by a breakup of prevalent coronary heart disease (CHD).

			Total Population	No CHD	CHD	*p*-Value
		*n*	*n =* 450	*n =* 350 (78%)	*n =* 100 (22%)	
Patient characteristics						
	Age, years	450	63 ± 9	62 ± 9	66 ± 7	<0.001
	Male, *n* (%)	450	261 (58)	190 (54)	71 (71)	0.003
	Diabetes duration, years	450	11 (7–18)	11 (7–17)	13 (7–20)	0.15
	Systolic blood pressure, mmHg	449	136 ± 16	136 ± 16	136 ± 19	0.81
	Diastolic blood pressure, mmHg	449	74 ± 9	75 ± 9	72 ± 10	0.01
	Heart rate, beats/min	444	74 ± 13	75 ± 13	69 ± 11	<0.001
	Body surface area, m^2^	448	2.10 ± 0.22	2.10 ± 0.23	2.07 ± 0.19	0.27
	Urinary creatinine excretion, µmol/24 h	446	13.8 ± 4.8	13.8 ± 5.0	13.8 ± 4.2	0.97
Complications						
	Cerebrovascular disease, *n* (%)	450	47 (11)	87 (22)	13 (27)	0.44
	Peripheral artery disease, *n* (%)	450	44 (10)	80 (20)	20 (44)	<0.001
	Retinopathy, *n* (%)	447	106 (24)	78 (23)	32 (32)	0.05
	Neuropathy, *n* (%)	450	157 (36)	116 (33)	46 (46)	0.02
	Diabetic nephropathy, *n* (%)	446	183 (42)	131 (38)	58 (58)	<0.001
	eGFR < 60 mL/min·1.73 m^2^	450	101 (23)	74 (21)	30 (30)	0.06
	Microalbuminuria, *n* (%)	445	131 (30)	92 (27)	44 (44)	0.001
Lifestyle						
	Body mass index, kg/m^2^	448	32.8 ± 6.2	33.1 ± 6.4	32.1 ± 5.6	0.15
	Body mass index ≥ 30 kg/m^2^, *n* (%)	448	290 (65)	233 (67)	57 (58)	0.12
	Smoking, former or current, *n* (%)	450	306 (70)	235 (67)	78 (78)	0.04
	Alcohol	424				
	No alcohol, *n* (%)		148 (36)	123 (37)	32 (34)	0.80
	0–13 units per week, *n* (%)		206 (50)	159 (48)	49 (52)	
	≥14 units per week, *n* (%)		61 (15)	47 (14)	14 (15)	
	Adherence guideline physical activity, *n* (%)	433	249 (59)	201 (60)	52 (54)	0.34
Pharmacological treatment						
	Insulin use, *n* (%)	450	275 (63)	218 (62)	68 (68)	0.30
	Statin use, *n* (%)	450	331 (76)	254 (73)	86 (86)	0.006
	Beta blocker treatment, *n* (%)	450	202 (46)	131 (37)	77 (77)	<0.001
	RAAS inhibition, *n* (%)	450	289 (66)	225 (64)	73 (73)	0.10
	Calcium antagonists, *n* (%)	450	98 (22)	66 (19)	36 (36)	<0.001
	Thiazide diuretics, *n* (%)	450	136 (31)	108 (31)	29 (29)	0.72
	Loop diuretics, *n* (%)	450	75 (17)	48 (14)	33 (33)	<0.001
	Number of antihypertensives	450	2 (1–3)	2 (1–3)	3 (2–4)	<0.001
Magnesium parameters						
	Dietary magnesium intake *, mg/day	438	305 ± 46	309 ± 47	292 ± 40	0.001
	Urinary magnesium excretion, mmol/24 h	402	3.94 ± 2.05	4.03 ± 2.05	3.66 ± 2.02	0.13
	Plasma magnesium concentration, mmol/L	432	0.77 ± 0.09	0.78 ± 0.08	0.76 ± 0.09	0.06
	Hypomagnesemia, *n* (%)	432	73 (17)	53 (16)	20 (20)	0.35
Serum values						
	Total cholesterol, mmol/L	447	4.0 ± 0.9	4.1 ± 0.9	3.8 ± 1.1	0.04
	HDL cholesterol, mmol/L	445	1.1 ± 0.3	1.2 ± 0.4	1.0 ± 0.3	<0.001
	LDL cholesterol, mmol/L	428	2.0 ± 0.7	2.0 ± 0.7	1.9 ± 0.8	0.25
	HbA1c, mmol/mol	448	57 ± 12	57 ± 12	58 ± 12	0.43
Dietary intake						
	Total energy intake, kcal/day	438	1922 ± 629	1904 ± 649	1932 ± 630	0.71
	Urinary sodium excretion, mmol/24 h	444	185 ± 79	183 ± 67	197 ± 84	0.14
	Urinary potassium excretion, mmol/24 h	439	77 ± 25	78 ± 26	77 ± 21	0.87
	Calcium intake, mg/day	438	969 ± 441	979 ± 467	905 ± 358	0.16
	Fiber intake, g/day	438	20.9 ± 6.6	20.8 ± 7.0	20.4 ± 6.1	0.60
	Cholesterol, g/day	438	194 ± 96	195 ± 101	188 ± 79	0.51
	Total fat intake, g/day	438	79 ± 39	78 ± 34	81 ± 34	0.52
	Total protein intake, g/day	438	79 ± 23	79 ± 24	76 ± 22	0.18
	Total carbohydrate intake, g/day	438	207 ± 69	205 ± 72	209 ± 67	0.61

CHD: coronary heart disease, eGFR: estimated glomerular filtration rate (CKD-EPI), HDL: high density lipoprotein, LDL: low density lipoprotein, HbA1c: glycated hemoglobin. * Dietary magnesium intake was adjusted for total energy intake using the residual method.

**Table 2:** Prevalence ratios (95% CI) for associations between dietary, urinary and plasma Magnesium and coronary heart disease in type 2 diabetes from the DIAbetes and LifEstyle Cohort Twente (DIALECT) (*n* = 450).

	Quartile 1	Quartile 2	Quartile 3	Quartile 4	*p*-Trend
Dietary Mg intake *, mg/day	254 ± 25	291 ± 7	315 ± 8	361 ± 39	
*n* cases/*n* total	33/109	25/110	23/110	13/109	
Model 1 ^a^	1.00	0.71 (0.42, 1.22)	0.64 (0.37, 1.10)	0.40 (0.20, 0.77)	0.005
Model 2 ^b^	1.00	0.72 (0.42, 1.23)	0.69 (0.40, 1.21)	0.42 (0.22, 0.82)	0.01
Model 3 ^c^	1.00	0.71 (0.41, 1.23)	0.72 (0.41, 1.27)	0.40 (0.20, 0.79)	0.01
Urinary Mg excretion, mmol/24 h	1.81 ± 0.63	3.05 ± 0.32	4.32 ± 0.57	6.64 ± 1.75	
*n* cases/*n* total	24/101	24/100	19/101	15/100	
Model 1 ^a^	1.00	0.95 (0.54, 1.67)	0.73 (0.39, 1.35)	0.63 (0.33, 1.19)	0.24
Model 2 ^b^	1.00	1.28 (0.71, 2.30)	0.96 (0.51, 1.82)	0.74 (0.39, 1.42)	0.33
Model 3 ^c^	1.00	1.27 (0.70, 2.30)	0.85 (0.44, 1.65)	0.63 (0.32, 1.26)	0.13
Plasma Mg concentration, mmol/L	0.67 ± 0.06	0.75 ± 0.02	0.80 ± 0.02	0.88 ± 0.04	
*n* cases/*n* total	29/113	22/106	27/111	16/102	
Model 1 ^a^	1.00	0.91 (0.52, 1.60)	1.03 (0.60, 1.77)	0.60 (0.31, 1.14)	0.15
Model 2 ^b^	1.00	0.91 (0.51, 1.62)	1.09 (0.63, 1.89)	0.58 (0.30, 1.12)	0.17
Model 3 ^c^	1.00	0.91 (0.51, 1.63)	1.12 (0.65, 1.94)	0.62 (0.32, 1.20)	0.26

^a^ Model 1: Crude model ^b^ Model 2: Adjusted for age (years), BMI (kg/m^2^), smoking (never, former or current), alcohol consumption (none, 1–13 units per week, ≥14 units per week), physical activity (not adherent to guideline, adherent to guideline).^c^ Model 3: Model 2 + Total energy intake (kcal), 24 h urinary sodium excretion (mmol/24 h) and 24 h urinary potassium excretion (mmol/24 h).* Dietary magnesium intake was adjusted for total energy intake using the residual method.

**Table 3:** Prevalence ratios (95% CI) for associations between magnesium intake from different food sources in type 2 diabetes patients from the DIALECT cohort (*n* = 450).

	Model 1 ^a^	Model 2 ^b^	Model 3 ^c^
Source of magnesium intake	PR (95% CI)	PR (95% CI)	PR (95% CI)
Magnesium intake from cereals *, 10 mg/day	1.02 (0.94, 1.10)	1.02 (0.94, 1.10)	0.95 (0.86, 1.05)
Magnesium intake from dairy *, 10 mg/day	0.95 (0.87, 1.03)	0.95 (0.87, 1.03)	0.92 (0.84, 1.01)
Magnesium intake from coffee *, 10 mg/day	0.95 (0.83, 1.06)	0.95 (0.83, 1.08)	0.96 (0.84, 1.10)
Magnesium intake from potatoes *, 10 mg/day	1.03 (0.87, 1.22)	1.02 (0.86, 1.21)	0.97 (0.80, 1.16)
Magnesium intake from meat *, 10 mg/day	0.91 (0.70, 1.20)	0.91 (0.69, 1.19)	0.80 (0.59, 1.09)
Magnesium intake from legumes & nuts *, 10 mg/day	0.96 (0.89, 1.05)	0.96 (0.88, 1.06)	0.95 (0.86, 1.05)
Magnesium intake from fruit *, 10 mg/day	1.00 (0.81, 1.23)	0.98 (0.79, 1.20)	0.96 (0.78, 1.19)
Magnesium intake from vegetables *, 10 mg/day	0.71 (0.51, 1.01)	0.71 (0.50, 1.01)	0.75 (0.52, 1.08)
Magnesium intake from miscellaneous sources *, 10 mg/day	0.95 (0.89, 1.02)	0.95 (0.89, 1.03)	0.90 (0.82, 0.99)

^a^ Model 1: Crude model ^b^ Model 2: Adjusted for age (years), BMI (kg/m^2^), smoking (never, former/current), alcohol consumption (none, 1–13 units per week, ≥14 units per week), physical activity (not adherent to guideline, adherent to guideline).^c^ Model 3: Model 2 + Total energy intake (kcal), magnesium intake from the other sources (cereals (mg/day), dairy (mg/day), coffee (mg/day), potatoes (mg/day), meat (mg/day), legumes and nuts (mg/day), fruit (mg/day), vegetables (mg/day), and other (mg/day)). * Magnesium intake from food sources was adjusted for total energy intake using the residual method. An increment of 10 mg magnesium intake per day was used to calculate PR.PR, prevalence ratio; CI, confidence interval.

## Supplementary Materials

The following are available online at http://www.mdpi.com/2072-6643/10/3/307/s1, Table S1: Overview of food items included in each food category, Table S2: Baseline characteristics of patients with T2D by a breakup of dietary magnesium intake.
