# Supplementary material for: Higher Dietary Magnesium Intake and Higher Magnesium Status Are Associated with Lower Prevalence of Coronary Heart Disease in Patients with Type 2 Diabetes
Source: Nutrients. 2018 Mar 5;10(3):307. doi: 10.3390/nu10030307 (PMC5872725; doi:10.3390/nu10030307)
Supplement: Supplementary file 1 [file nutrients-10-00307-s001.zip › Supplementary Table S1.docx]

**Supplementary Table S1.** Overview of food items included in each food category

| **1** | **Cereals** | **3** | **Coffee** |
| --- | --- | --- | --- |
|  | Bread |  | Coffee |
|  | Rye bread | **4** | **Potatoes** |
|  | Raisin bread |  | Boiled/mashed potatoes without fat |
|  | Bread rolls |  | Fried/mashed potatoes with fat |
|  | Croissants |  | Oven-grilled french fries |
|  | Rusk |  | Deep fried french fries |
|  | Pasta |  | French fries prepared by someone else |
|  | Rice | **5** | **Meat** |
| **2** | **Dairy** |  | Chicken, turkey |
|  | Full milk |  | Beef, steak etc. |
|  | Semi-skimmed milk |  | Beef, blind finch, etc. |
|  | Skimmed milk |  | Pork, steak etc. |
|  | Buttermilk |  | Pork, chop etc. |
|  | Other milk, such as horse and goat |  | Pork, smoked sausage etc. |
|  | Normal milk in coffee |  | Lamb or mutton |
|  | Coffee milk, coffee cream |  | Other meats such as goat/horse |
|  | Full fat milk beverages |  | Cooked liver |
|  | Semi-skimmed milk beverages |  | Hepatic/renal products |
|  | Skimmed milk beverages |  | Sausages as in between snack |
|  | Beverages with probiotics |  | Minced meat (all types) |
|  | Unknown dairy drink |  | Liver products |
|  | Unknown type of milk |  | Ham etc. |
|  | Full (fruit) yogurt |  | Lunch meats |
|  | Semi-skimmed (fruit) yogurt |  | Bacon etc. |
|  | Skimmed (fruit) yogurt |  | Unknown type of meat |
|  | Yogurt with probiotics | **6** | **Legumes and nuts** |
|  | Cholesterol-lowering yoghurt drink |  | Legumes |
|  | Full custard & pudding |  | Split peas, beans etc. |
|  | Low-fat custard & pudding |  | Split pea soup |
|  | Lean custard & pudding |  | Nuts and seeds |
|  | 20+/30+ cheese |  | Peanuts (and coated peanuts) |
|  | 40+ cheese |  | Nuts as in between snack |
|  | Ordinary cheese (48+) |  | Soy products |
|  | Luxury fat cheese | **7** | **Fruit** |
|  | Low-fat cheese |  | Citrus fruit |
|  | Unknown type of cheese |  | Other fruit |
|  | Cheese used in hot meal |  | Fruit in can/jar |
|  | Cheese as in between snack | **8** | **Vegetables** |
|  | Full (fruit) cream cheese |  | Broccoli, cauliflower etc. |
|  | Semi-skimmed (fruit) cream cheese |  | Sal, spinach, endive etc. |
|  | Skimmed (fruit) cream cheese |  | Mushrooms |
|  | Ice cream |  | Onion, pepper, etc |
|  | Whipped cream |  | Raw vegetables |
|  | Cream used in hot meals | **9** | **Miscellaneous** |
|  | Hot porridge |  | Beverages |
|  | Breakfast drink |  | Warm snacks |
|  | Butter |  | Cold snacks |
|  | Diet butter |  | Sauces |
|  |  |  | Fish |
|  |  |  | Peanut butter |
|  |  |  | Jam |
|  |  |  | Chocolate Paste |
