# Supplementary material for: Higher Dietary Magnesium Intake and Higher Magnesium Status Are Associated with Lower Prevalence of Coronary Heart Disease in Patients with Type 2 Diabetes
Source: Nutrients. 2018 Mar 5;10(3):307. doi: 10.3390/nu10030307 (PMC5872725; doi:10.3390/nu10030307)
Supplement: Supplementary file 1 [file nutrients-10-00307-s001.zip › Supplementary Table S2.docx]

**Supplementary Table S2.** Baseline characteristics of patients with T2D by a breakup of dietary magnesium intake

|  | | |  | Sex-specific quartiles of energy adjusted magnesium intake | | | |  |
| --- | --- | --- | --- | --- | --- | --- | --- | --- |
|  | | |  | Quartile 1 | Quartile 2 | Quartile 3 | Quartile 4 |  |
| *Mean adjusted magnesium intake* | | | *305 ± 46* | *254 ± 25* | *291 ± 7* | *315 ± 8* | *361 ± 39* |  |
| *Variable* | | | *n=450* | *109* | *110* | *110* | *109* |  |
|  | Age, years | | 63 ± 9 | 63 ± 9 | 64 ± 9 | 63 ± 8 | 63 ± 10 | 0.86 |
|  | Female, n (%) | | 186 (43) | 46 (42) | 47 (43) | 47 (43) | 46 (42) | n.a. |
|  | Diabetes duration, years | | 11 [7-18] | 11 [6-18] | 12 [7-19] | 11 [6-18] | 11 [7-16] | 0.68 |
|  | Systolic blood pressure, mmHg | | 136 ± 16 | 137 ± 17 | 139 ± 18 | 137 ± 16 | 133 ± 13 | 0.05 |
|  | Diastolic blood pressure, mmHg | | 74 ± 9 | 75 ± 10 | 74 ± 9 | 76 ± 10 | 74 ± 9 | 0.50 |
|  | Heart rate, beats/min | | 74 ± 13 | 74 ± 13 | 73 ± 14 | 74 ± 12 | 75 ± 12 | 0.60 |
|  | Body surface area, m^2^ | | 2.10 ± 0.22 | 2.11 ± 0.24 | 2.09 ± 0.21 | 2.08 ± 0.21 | 2.11 ± 0.21 | 0.67 |
|  | Urinary creatinine excretion, µmol/24h | | 13.8 ± 4.8 | 14.1 ± 5.6 | 13.8 ± 4.9 | 13.5 ± 4.1 | 13.8 ± 4.5 | 0.84 |
| *Complications* | | |  |  |  |  |  |  |
|  | Coronary heart disease, n (%) | | 100 (22) | 33 (31) | 25 (23) | 23 (21) | 13 (12) | 0.007 |
|  | Cerebrovascular disease, n (%) | | 47 (11) | 14 (13) | 11 (10) | 10 (9) | 12 (11) | 0.83 |
|  | Peripheral artery disease, n (%) | | 44 (10) | 16 (15) | 10 (9) | 7 (6) | 11 (10) | 0.23 |
|  | Retinopathy, n (%) | | 106 (24) | 28 (26) | 38 (35) | 17 (16) | 23 (21) | 0.007 |
|  | Neuropathy, n (%) | | 157 (36) | 38 (35) | 40 (36) | 36 (33) | 43 (39) | 0.77 |
|  | Diabetic nephropathy, n (%) | | 183 (42) | 51 (47) | 49 (45) | 44 (40) | 39 (36) | 0.36 |
|  |  | eGFR<60 | 101 (23) | 33 (30) | 25 (23) | 24 (22) | 19 (17) | 0.16 |
|  |  | Microalbuminuria, n (%) | 131 (30) | 33 (31) | 39 (36) | 30 (27) | 29 (27) | 0.42 |
| *Lifestyle* | | |  |  |  |  |  |  |
|  | BMI, kg/m2 | | 32.8 ± 6.2 | 33.2 ± 6.5 | 33.3 ± 6.3 | 32.5 ± 6.5 | 32.2 ± 5.5 | 0.49 |
|  | Smoking, former or current, n (%) | | 306 (70) | 76 (70) | 80 (73) | 73 (66) | 77 (71) | 0.78 |
|  | Alcohol | |  |  |  |  |  |  |
|  |  | No alcohol, n (%) | 148 (36) | 35 (34) | 34 (32) | 39 (38) | 40 (40) | 0.40 |
|  |  | 0-13 units per week, n (%) | 206 (50) | 46 (45) | 58 (55) | 52 (50) | 50 (49) |  |
|  |  | 14+ units per week, n (%) | 61 (15) | 22 (21) | 14 (13) | 13 (13) | 12 (12) |  |
|  | Adherence guideline physical activity, n (%) | | 249 (59) | 57 (54) | 64 (60) | 63 (58) | 65 (63) | 0.61 |
| *Pharmaceutical treatment* | | |  |  |  |  |  |  |
|  | Insulin use, n (%) | | 275 (63) | 65 (60) | 70 (64) | 65 (59) | 75 (69) | 0.42 |
|  | Statin use, n (%) | | 331 (76) | 83 (76) | 80 (73) | 90 (82) | 78 (72) | 0.29 |
|  | β-blocker treatment, n (%) | | 202 (46) | 60 (55) | 55 (50) | 46 (42) | 41 (38) | 0.04 |
|  | RAAS inhibition, n (%) | | 289 (66) | 74 (68) | 78 (71) | 68 (62) | 69 (63) | 0.46 |
|  | Calcium antagonists, n (%) | | 98 (22) | 27 (25) | 33 (30) | 21 (19) | 17 (16) | 0.06 |
|  | Thiazide diuretics, n (%) | | 136 (31) | 33 (30) | 42 (38) | 38 (35) | 23 (21) | 0.04 |
|  | Loop diuretics, n (%) | | 75 (17) | 22 (20) | 18 (16) | 16 (15) | 19 (17) | 0.73 |
|  | Number of antihypertensives | | 2 [1-3] | 2 [1-3] | 2 [1-3] | 2 [1-3] | 2 [0-3] | 0.008 |
| *Magnesium values* | | |  |  |  |  |  |  |
|  | Urinary magnesium excretion, mmol/24h | | 3.94 ± 2.05 | 3.94 ± 2.31 | 3.57 ± 1.79 | 3.67 ± 1.81 | 4.61 ± 2.14 | 0.002 |
|  | Plasma magnesium concentration, mmol/l | | 0.77 ± 0.09 | 0.77 ± 0.10 | 0.76 ± 0.09 | 0.77 ± 0.08 | 0.78 ± 0.08 | 0.20 |
|  | Hypomagnesemia, n (%) | | 72 (17) | 21 (20) | 25 (23) | 16 (16) | 10 (10) | 0.06 |
| *Serum values* | | |  |  |  |  |  |  |
|  | Total cholesterol, mmol/l | | 4.0 ± 0.9 | 4.0 ± 1.0 | 4.0 ± 0.9 | 3.9 ± 0.9 | 4.1 ± 1.0 | 0.82 |
|  | HDL cholesterol, mmol/l | | 1.1 ± 0.3 | 1.1 ± 0.4 | 1.1 ± 0.3 | 1.1 ± 0.3 | 1.2 ± 0.4 | 0.87 |
|  | LDL cholesterol, mmol/l | | 2.0 ± 0.7 | 2.1 ± 0.8 | 2.0 ± 0.7 | 2.0 ± 0.7 | 2.0 ± 0.8 | 0.86 |
|  | HbA1c, mmol/mol | | 57 ± 12 | 57 ± 12 | 56 ± 11 | 57 ± 11 | 59 ± 13 | 0.23 |
| *Dietary intake* | | |  |  |  |  |  |  |
|  | Total energy intake, kcal/day | | 1922 ± 629 | 2071 ± 665 | 1797 ± 661 | 1850 ± 562 | 1973 ± 594 | 0.006 |
|  | Urinary sodium excretion, mmol/24h | | 185 ± 79 | 177 ± 82 | 185 ± 79 | 174 ± 65 | 204 ± 88 | 0.03 |
|  | Urinary potassium excretion, mmol/24h | | 77 ± 25 | 70 ± 24 | 75 ± 25 | 78 ± 26 | 86 ± 24 | <0.001 |
|  | Calcium intake, mg/day | | 969 ± 441 | 993 ± 456 | 869 ± 441 | 982 ± 398 | 1150 ± 420 | <0.001 |
|  | Fiber intake, g/day | | 20.9 ± 6.6 | 19.4 ± 6.1 | 19.2 ± 6.6 | 21.1 ± 6.1 | 23.8 ± 6.6 | <0.001 |
|  | Cholesterol, g/day | | 194 ± 96 | 225 ± 126 | 193 ± 96 | 177 ± 75 | 183 ± 75 | 0.001 |
|  | Total fat intake, g/day | | 79 ± 39 | 89 ± 39 | 74 ± 32 | 73 ± 28 | 79 ± 33 | 0.001 |
|  | Total protein intake, g/day | | 79 ± 23 | 76 ± 24 | 73 ± 23 | 78 ± 21 | 87 ± 22 | <0.001 |
|  | Total carbohydrate intake, g/day | | 207 ± 69 | 217 ± 67 | 193 ± 76 | 206 ± 67 | 214 ± 63 | 0.05 |
